# Maleimide Self-Reaction in Furan/Maleimide-Based Reversibly Crosslinked Polyketones: Processing Limitation or Potential Advantage?

**DOI:** 10.3390/molecules26082230

**Published:** 2021-04-13

**Authors:** Felipe Orozco, Zafarjon Niyazov, Timon Garnier, Nicola Migliore, Alexander T. Zdvizhkov, Patrizio Raffa, Ignacio Moreno-Villoslada, Francesco Picchioni, Ranjita K. Bose

**Affiliations:** 1 Department of Chemical Engineering, Product Technology, University of Groningen, Nijenborgh 4, 9747 AG Groningen, The Netherlands; f.orozco.gutierrez@rug.nl (F.O.); z.niyazov@student.rug.nl (Z.N.); T.I.Garnier@student.rug.nl (T.G.); n.migliore@rug.nl (N.M.); azdvizhkov@gmail.com (A.T.Z.); p.raffa@rug.nl (P.R.); f.picchioni@rug.nl (F.P.); 2 Laboratorio de Polímeros, Instituto de Ciencias Químicas, Facultad de Ciencias, Universidad Austral de Chile, Casilla 567, Valdivia, Chile; imorenovilloslada@uach.cl

**Keywords:** Diels-Alder, thermo-reversibly crosslinked polymers, maleimide self-reaction, maleimide homopolymerization, self-healing polymers

## Abstract

Polymers crosslinked via furan/maleimide thermo-reversible chemistry have been extensively explored as reprocessable and self-healing thermosets and elastomers. For such applications, it is important that the thermo-reversible features are reproducible after many reprocessing and healing cycles. Therefore, side reactions are undesirable. However, we have noticed irreversible changes in the mechanical properties of such materials when exposing them to temperatures around 150 °C. In this work, we study whether these changes are due to the self-reaction of maleimide moieties that may take place at this rather low temperature. In order to do so, we prepared a furan-grafted polyketone crosslinked with the commonly used aromatic bismaleimide (1,1′-(methylenedi-4,1-phenylene)bismaleimide), and exposed it to isothermal treatments at 150 °C. The changes in the chemistry and thermo-mechanical properties were mainly studied by infrared spectroscopy, ^1^H-NMR, and rheology. Our results indicate that maleimide self-reaction does take place in the studied polymer system. This finding comes along with limitations over the reprocessing and self-healing procedures for furan/maleimide-based reversibly crosslinked polymers that present their softening (decrosslinking) point at relatively high temperatures. On the other hand, the side reaction can also be used to tune the properties of such polymer products via in situ thermal treatments.

## 1. Introduction

**Figure 1:**
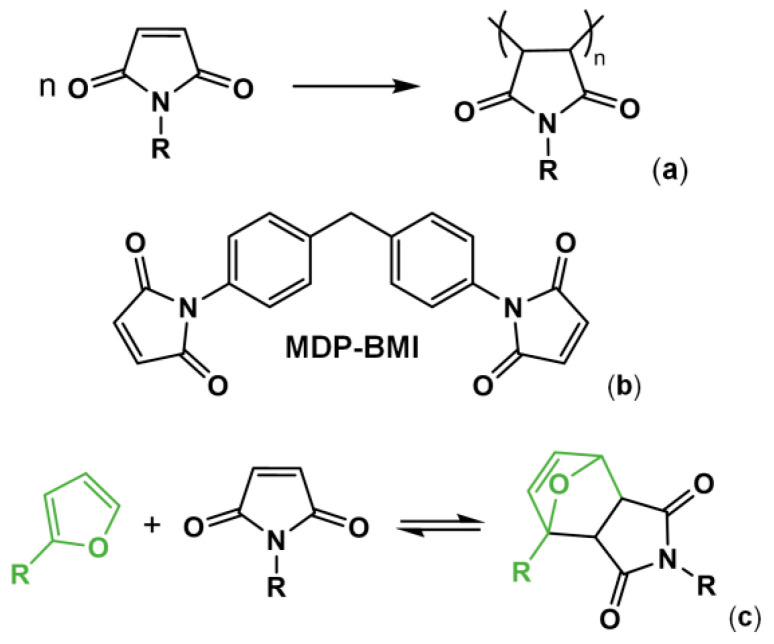
(**a**) Maleimide self-reaction into succinimide repetitive units. (**b**) MDP-BMI. (**c**) Diels-Alder (DA) thermo-reversible reaction between furan and maleimide.

Maleimide chemistry has long been used in the preparation of polymer resins [1,2,3,4]. At temperatures above 200 °C, maleimides undergo homopolymerization in bulk via free radicals, forming a backbone of succinimide units (Figure 1a) [2,3,5]. This self-reaction is also known to occur for 1,1′-(methylenedi-4,1-phenylene)bismaleimide (MDP-BMI) (Figure 1b) at lower temperatures (from 180 °C [2,5]) even without free-radical initiators [5]. However, such chemistry presents a drawback; it is irreversible. Therefore, reprocessing this sort of polymer product is overly complicated.

Nowadays, in the polymer field, MDP-BMI receives much more attention as a Diels-Alder (DA) dienophile, with furan as its diene counterpart (Figure 1c) [4,6,7,8,9,10,11,12,13,14]. This DA pair reacts thermo-reversibly, which allows for preparation of polymer networks with crosslinking points that cleave and form thermodynamically. The DA pair also presents fast kinetics and forms and cleaves at practical temperatures, at approximately 60 and 110 °C, respectively [8,14]. Over the last two decades, many furan/maleimide-based thermo-reversibly crosslinked polymers (FM-TRCP) have been explored as reprocessable thermosets and elastomers, and self-healing materials [6,7,8,9,10,11,12,13,14,15,16,17].

**Figure 2:**
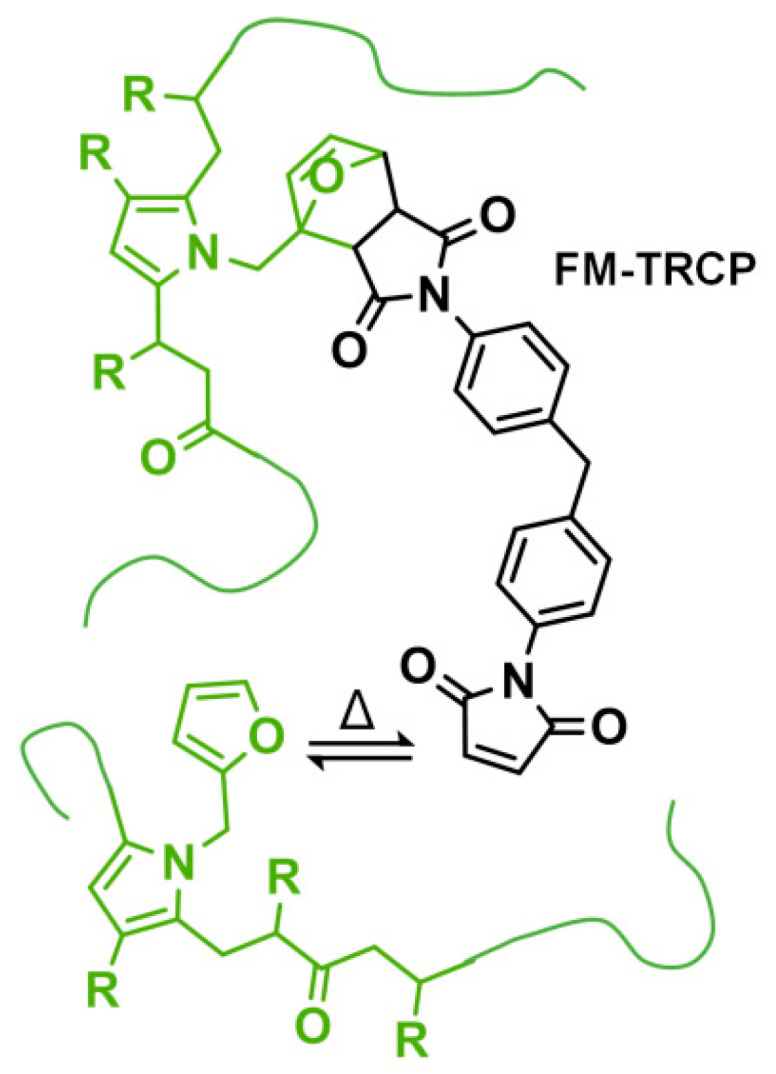
Prepared FM-TRCP: furan-grafted polyketone thermo-reversibly crosslinked with MDP-BMI. R stands for hydrogen and methyl groups.

The reversibility of these FM-TRCP is remarkable—for example, Zhang et al. prepared a polyketone thermoset that could be reprocessed many times without considerable changes in the mechanical properties [6]. However, while working with different FM-TRCP, we noticed that irreversible changes in the thermo-mechanical properties take place when handling the materials at high temperatures (140–170 °C). Here, we prepared a crosslinked MDP-BMI furan-grafted polyketone (Figure 2), and explored if this irreversible effect was caused by the MDP-BMI reacting with itself at these rather low temperatures. In order to do so, we studied the thermal stability of the system at 150 °C by FTIR, ^1^H-NMR, and rheology.

## 2. Results

**Figure 3:**
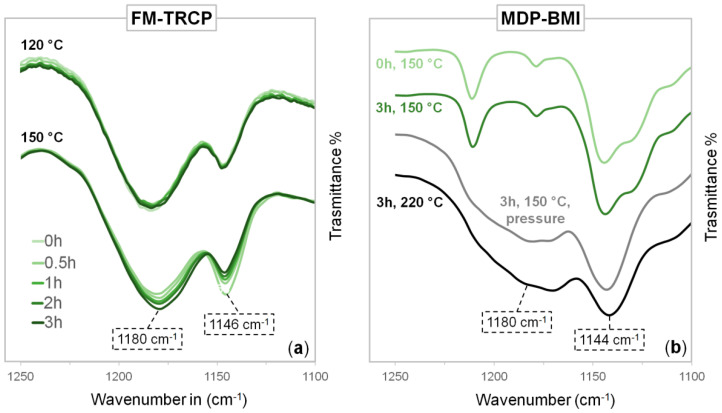
(**a**) FTIR measurements of the FM-TRCP over time at 120 and 150 °C. (**b**) FTIR measurements of MDP-BMI before and after 3 h at 150 °C, after being in a press for 3 h at 150 °C (4 MPa), and after 3 h at 220 °C.

FTIR spectra of the studied FM-TRCP were taken at isothermal conditions over three hours (Figure 3a). The spectra taken at the beginning of the measurements display the thermo-reversible equilibrium already displaced towards the reactants (free furan and maleimide groups), since the retro-DA kinetics are quite fast [8,14]. Figure 3a shows the peaks at 1180 and 1146 cm^−1^ corresponding to the CNC bending mode of the succinimide and maleimide rings, respectively [3,18,19,20]. When measured at 120 °C, there are no appreciable differences in these peaks through time. On the other hand, at 150 °C, there is a significant increase of the succinimide peak and a reduction of the maleimide one. Other peaks attributed to maleimide groups decrease as well—imide CH wagging at 827 cm^−1^ and the CH out-of-plane bending mode at 691 cm^−1^ [3,18,19] (Figure S1 from the Supplementary Materials).

Control experiments using MDP-BMI alone were also carried out by FTIR. Figure 3b shows no noticeable difference between the spectra taken before and after three hours at 150 °C. However, the strong signal attributed to the succinimide rings, appears at 1180 cm^−1^ after heating MDP-BMI for three hours under pressure at 150 °C and 4 MPa (using a press), and when heated at vacuum to 220 °C also for three hours.

**Figure 4:**
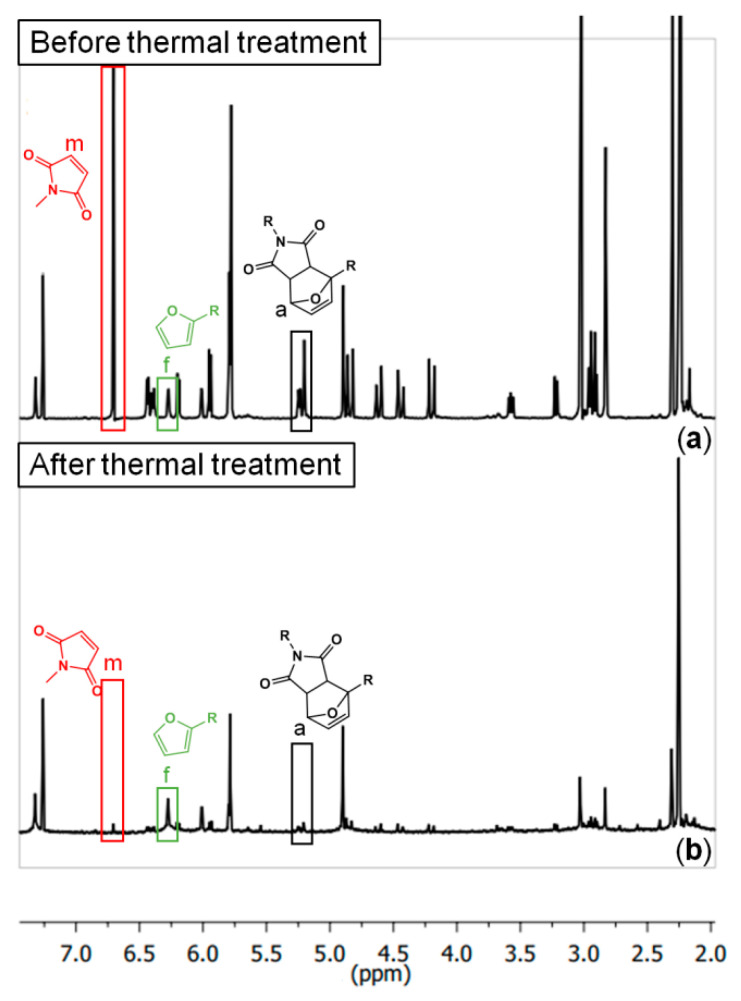
^1^H-NMR spectra of the DA-adduct model system (**a**) before and (**b**) after three hours at 150 °C (full interpretation in [21]).

A model DA-adduct (shown in Table 1) was prepared in order to explore the system by ^1^H-NMR (Figure 4). Samples of the model were taken before and after being exposed to 150 °C for three hours, and were dissolved in d-chloroform in order to be measured. However, after the thermal treatment, the model system was not fully soluble (in all common solvents that were tried: DFM, toluene, acetone, methanol, DMSO, and THF). Therefore, the spectrum showed in Figure 4b is only from the soluble fraction after the thermal treatment. The percentages of the species seen in the NMR spectra are tabulated in Table 1. As expected, after the thermal treatment, the model DA system is mostly decoupled. However, there is almost no free maleimide in the system.

**Figure 5:**
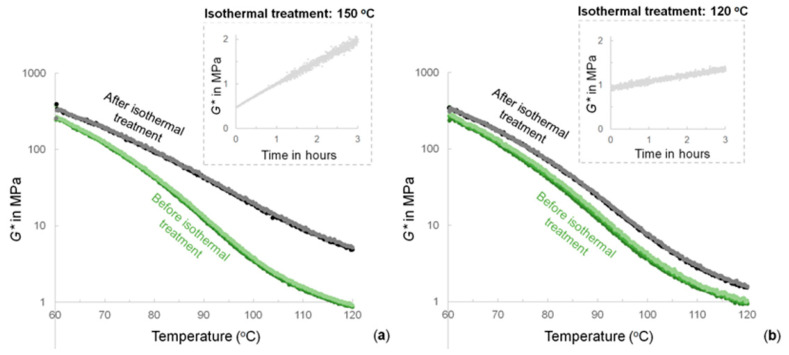
Thermo-mechanical profiles of the FM-TRCP before (three heating profiles in different tones of green) and after (two heating profiles in black and gray) isothermal treatments at (**a**) 150 °C and (**b**) 120 °C (insets). For simplicity, only the heating profiles are shown.

The rheological measurements are shown in Figure 5. The FM-TRCP was submitted to several heating and cooling cycles under oscillatory strain. After two cycles, the FM-TRCP was set to 150 °C and kept under isothermal conditions for 3 h. Subsequently, two additional cycles were performed. Figure 5 shows the drop of the storage modulus (G*) due to the decreasing crosslinking density of the polymer as the temperature increases in each cycle. There is also a clear change in the mechanical properties after the isothermal treatment at 150 °C (Figure 5a). During this isothermal step, G* increases notably (inset figure). The same procedure was also carried out, using 120 °C during the isothermal treatment (Figure 5b). As a result, the mechanical profiles of the FM-TRCP only changed slightly after the isothermal treatment. Additionally, Figure 5 shows the remarkable reproducibility of the thermo-reversible features of the system, evidenced by the overlapping heating profiles carried out, both before and after holding the temperature at isothermal conditions. As a control experiment, the change in the mechanical properties of MDP-BMI alone at 150 °C was also monitored (G* doubled in 80 min—Figure S2 from the Supplementary Materials).

## 3. Discussion

FTIR control experiments over MDP-BMI (Figure 3b), before and after being exposed to 220 °C, showed the expected peak at 1180 cm^−1^ related to the succinimide rings that are formed as the maleimide groups self-react [3]. The peaks from the reacting unsaturated carbons =CH- around 3100 cm^−1^ also decrease (Figure S3 from the Supplementary Materials) [22]. However, when MDP-BMI is exposed to isothermal treatments at 150 °C, these changes in the spectra are only seen if the bismaleimide is set under pressure. This might be due to mobility issues, as MDP-BMI is solid at this temperature and might not be able to self-react without pressure. This is supported by observations of MDP-BMI solutions in DMSO (where the bismaleimide molecules have no mobility restrictions) that show swollen precipitates after a couple of hours at 150 °C. Similarly, rheology experiments of MDP-BMI at 150 °C, which were performed under constant axial force, also showed a clear change in the mechanical properties of the bismaleimide over time.

In the experiments using the FM-TRCP at 150 °C (Figure 3a), the peak from the maleimide groups (1146 cm^−1^) decreases over time, while the succinimide one (1180 cm^−1^) increases. This latter peak is already seen in the very first measurement (0 h), since there are still DA adducts present at this temperature and the adduct has a succinimide ring in its structure (Figure 1c). At this temperature, as time goes on, the free maleimide groups gradually seem to self-react and form additional succinimide rings in the system, though these ones are formed irreversibly (Figure 1a). Interestingly, even though the FM-TRCP presents a much lower density of maleimide moieties than pure MDP-BMI, the self-reaction does take place at 150 °C in the polymer system without applying pressure. This might be because the polymer is already at about 30 °C above its softening temperature, so that the maleimide moieties have much more mobility than in MDP-BMI that is solid at this temperature.

The results obtained by ^1^H-NMR using the model DA-adduct show that the maleimide groups are practically absent in the spectra after the thermal treatment (Table 1, Figure 4). This suggests that the free maleimides are transforming into something else. However, these newly formed species (probably succinimide oligomers) are not possible to identify via solution NMR due to their poor solubility. Methyl maleimide molecules could also be leaving the system through evaporation. However, it is unlikely that a considerable fraction is being lost this way, since the temperature of the thermal treatment is much lower than the boiling point of methyl maleimide (boiling temperature above 200 °C).

Next, other possible chemical changes are taken into consideration, such as the decomposition of certain moieties. However, a thermo-gravimetric analysis, performed under air, suggests otherwise. After an hour at 150 °C, the FM-TRCP loses less than 2% of its weight (Figure S4 from the Supplementary Materials). Such small weight loss can be attributed to the evaporation of residual solvent trapped within the polymer network from its synthesis. Even more, the FTIR spectra taken before and after the isothermal treatments differ only in the signals attributed to maleimide and succinimide. Therefore, the polymer does not seem to decompose under these conditions. Another possible scenario is the aromatization of the furan/maleimide adduct through dehydration. However, this is normally achieved by using an anhydride that helps the dehydration process [8,23]. We tested this possibility through ^1^H-NMR. However, the spectrum of the furan/maleimide model system, exposed to 150 °C for several hours, does not show any signs of aromatization (no phthalimide peaks between 7 and 8 ppm [24]) (Figure S5 from the Supplementary Materials). A non-chemical change is also taken into account, that is, phase separation of bismaleimide. Nevertheless, this seems unlikely given that the FTIR spectra do show a chemical change. Additionally, the system works as a covalent adaptable network [25]; that is, the crosslinker is not free in the matrix at high temperatures, but shifts between the available furan moieties. Therefore, the bismaleimide is not loose in the matrix, and it is unlikely that it will undergo phase separation.

Figure 5a shows how the thermo-mechanical profiles of the FM-TRCP clearly change after the thermal treatment at 150 °C. This suggests an important change in the polymer system and supports the former results obtained by spectroscopy. On the other hand, 120 °C seems to be a temperature low enough to avoid these important changes on the FM-TRCP system, as seen in the rheology measurements using this temperature for the isothermal treatment (Figure 5b) and in the FTIR measurements that were run at 120 °C as well (Figure 3a).

**Figure 6:**
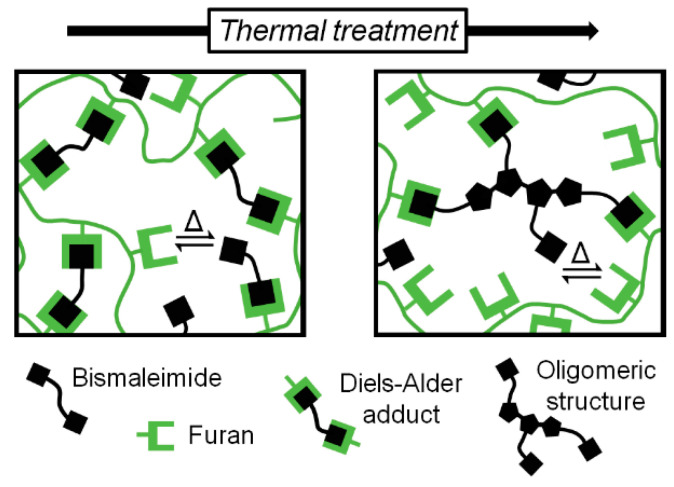
Polymer architecture before and after inducing maleimide self-reaction through heat.

Given that the polymer still shows some degree of crosslinking at 150 °C, thus having some mobility restrictions, the maleimide self-reaction is only expected to occur partially [3]. It is most likely that true homopolymerization of maleimide groups does not take place. Instead, the bismaleimide probably turns into oligomeric crosslinking structures that still contain active maleimide moieties (Figure 6). As shown in Figure 5a, this resulting arrangement comes along with considerable changes in the thermo-mechanical properties of the material without jeopardizing its thermo-reversibility.

Here, we present evidence indicating that the prepared polyketone-based FM-TRCP undergoes maleimide self-reaction at a rather low temperature (150 °C) even without adding any free-radical initiator. However, other polymer frameworks and bismaleimide species should be explored to address the universality of this effect. This finding impacts the design of reprocessing and self-healing procedures of FM-TRCP. For systems where the thermo-reversibility is displayed at relatively low temperatures, this maleimide self-reaction should not pose much of a problem, since it can be easily avoided. For instance, the polyketone system used in this work did not show any sign of the side reaction after 3 h at 120 °C (Figure 3a). At this temperature, G* had already reached a plateau at its minimum (Figure 5); thus, re-processing and self-healing procedures can be performed up to this temperature without expecting any side reaction. However, special attention is required for FM-TRCP with high softening temperatures. For example, Polgar et al. prepared an EPM rubber-based system, of which the softening point was around 170 °C [9]. For such materials, maleimide self-reaction might be unavoidable. Nonetheless, retro-DA takes place much faster than self-reacting maleimides; thus, the extent of the latter can be minimized through kinetic control.

The maleimide self-reaction can also be taken as an advantage. The reaction can be deliberately used to modify the thermo-mechanical performance of devices based on FM-TRCP. In this scenario, the irreversible process can be easily induced via an in situ thermal treatment without compromising the thermo-reversible features of the materials. Thus, the strategy would allow for synthesis of reprocessable and self-healing polymer products in which the thermo-mechanical performance can be tuned in situ. For instance, as seen in Figure 5a, the 3 h thermal treatment causes a substantial change in the mechanical properties, especially at higher temperatures where the G* increases by approximately an order of magnitude.

## 4. Materials and Methods

The furan-grafted polyketone crosslinked with MDP-BMI was prepared as previously described by Toncelli et al. [7]. The polymer was formulated using an equimolar ratio of furan and maleimide groups, and targeting 0.6 mmols of crosslinker per gram of the crosslinked polymer. The full characterization of this polyketone system can be found in the following reports [6,7,10,11].

An infrared spectrophotometer (Shimadzu IRTracer, Shimadzu Corporation, Kyoto, Japan), with attenuated total reflection and temperature control (Specac), was used to study the prepared FM-TRCP (ground into fine powder). The samples were studied under isothermal conditions for three hours at 150 and 120 °C. MDP-BMI alone was also measured. All MDP-BMI measurements were performed at 150 °C: one was measured as soon as the bismaleimide was placed in the spectrophotometer, another after 3 h at 150 °C, another also after 3 h at 150 °C but under 4 MPa of pressure (using a hot press), and one after being in a vacuum oven for 3 h at 220 °C.

The model DA-adduct shown in Table 1 was prepared as described by Zhang et al. [6]. The model was then set in a vial and exposed to 150 °C for 3 h. Before and after this thermal treatment, samples of the model system were taken and dissolved in d-chloroform. ^1^H-NMR measurements of these solutions were performed (NMR Oxford AS400, Oxford Instruments, Concord, MA, USA). The FM-TRCP was ground and molded into discs (8 mm diameter and 1 mm thick) at 120 °C and 4 MPa for 20 min. The thermo-mechanical properties of the discs were studied using a rheometer (Discovery HR-2, TA Instruments). The experiments were performed in oscillation mode (1 Hz) with 0.01% of strain, 8 N of axial force, and temperature ramps of 3 K/min. In order to erase the polymer thermal history, the measurements were started at 120 °C and followed by a cooling ramp down to 60 °C (below this temperature, the quality of measurements gets compromised as the samples start to slip from the measuring geometry). Then, two heating and cooling cycles were performed between 60 and 120 °C. Subsequently, the samples were heated up to 150 °C (or 120 °C for the control experiment), kept there for three hours, and then cooled down to 60 °C. Finally, two last heating–cooling cycles were set between 60 and 120 °C. These were performed using strain rates within the linear viscoelastic region (amplitude sweeps are shown in [21]).

Preliminary experiments of this work suggested 3 h as an adequate time to observe notable changes in the mechanical properties and infrared spectra without damaging the polymer. Therefore, all thermal treatments in this work were set to 3 h.

The experiments described above were performed in duplicate. All duplicates show reproducible spectroscopic and rheological measurements ([21]).

## Figures and Tables

**Table 1:** Percentages of the model DA mixture obtained by ^1^H-NMR before and after 3 h at 150 °C.

Model DA Mixture	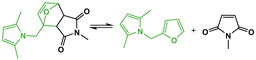		
Before (%)	49 ± 4	17 ± 1	34 ± 3
After (%)	28 ± 3	68 ± 4	3.5 ± 0.3

## Data Availability

The raw and processed data are available online at http://dx.doi.org/10.17632/wybdsgd59r.1.

## Supplementary Materials

The following are available online, Figure S1: FTIR measurements for the FM-TRCP performed at (**a**) 150 °C and (**b**) 120 °C, Figure S2: Increasing complex modulus of MDP-BMI at 150 °C, Figure S3: FTIR full spectra of MDP-BMI before and after 3 h at 150 °C, after being in a press for 3 h at 150 °C (4 MPa), and after 3 h at 220 °C, Figure S4: Thermo-gravimetric analysis of the polymer, Figure S5: H^1^-NMR from 8 to 7 ppm of the model compound (**a**) before and (**b**) after the thermal treatment at 150 °C.

## Sample Availability

Samples of the polyketone-based FM-TRCP are available from the authors.
