# Supplementary material for: Maleimide Self-Reaction in Furan/Maleimide-Based Reversibly Crosslinked Polyketones: Processing Limitation or Potential Advantage?
Source: Molecules. 2021 Apr 13;26(8):2230. doi: 10.3390/molecules26082230 (PMC8069175; doi:10.3390/molecules26082230)
Supplement: Supplementary file 1 [file molecules-26-02230-s001.pdf]

Supplementary figures

Maleimide self-reaction in furan/maleimide-based reversibly crosslinked polyketones: Processing limitation or potential advantage?

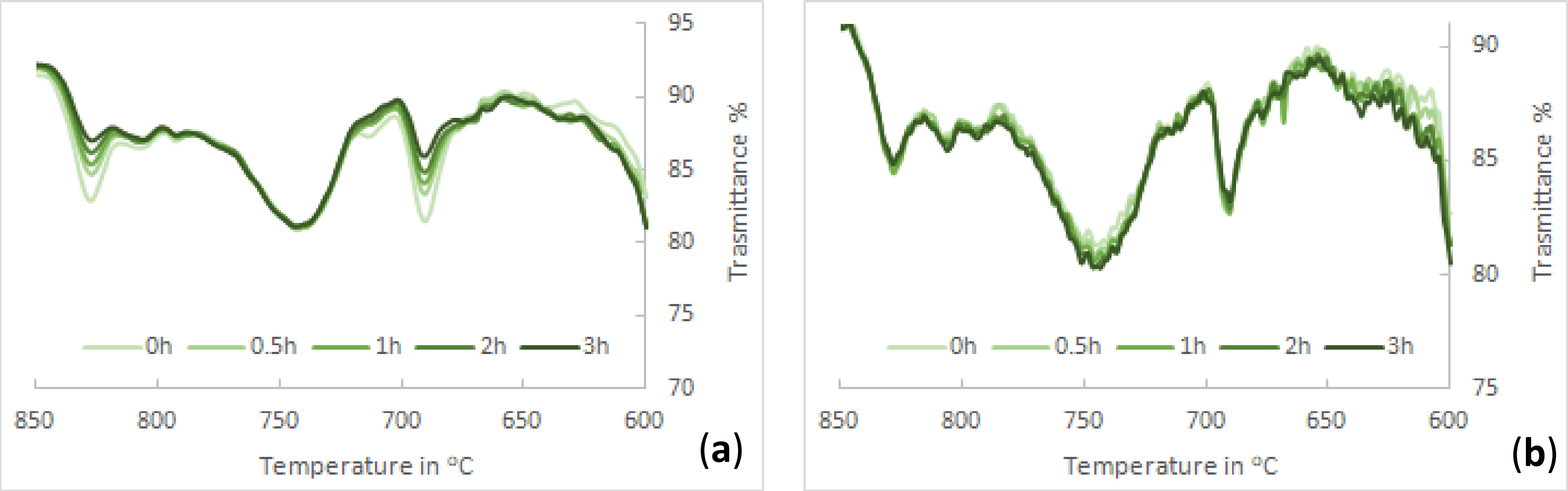

Figure S1. FTIR measurements for the FM-TRCP performed at (**a**) 150 °C and (**b**) 120 °C.

Supplementary figures

Maleimide self-reaction in furan/maleimide-based reversibly crosslinked polyketones: Processing limitation or potential advantage?

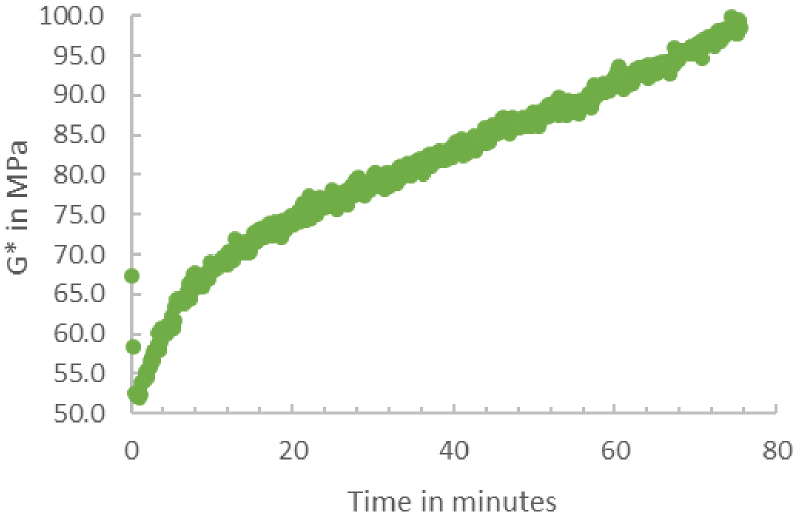

Figure S2. Increasing complex modulus of MDP-BMI at 150 °C.

Supplementary figures

Maleimide self-reaction in furan/maleimide-based reversibly crosslinked polyketones: Processing limitation or potential advantage?

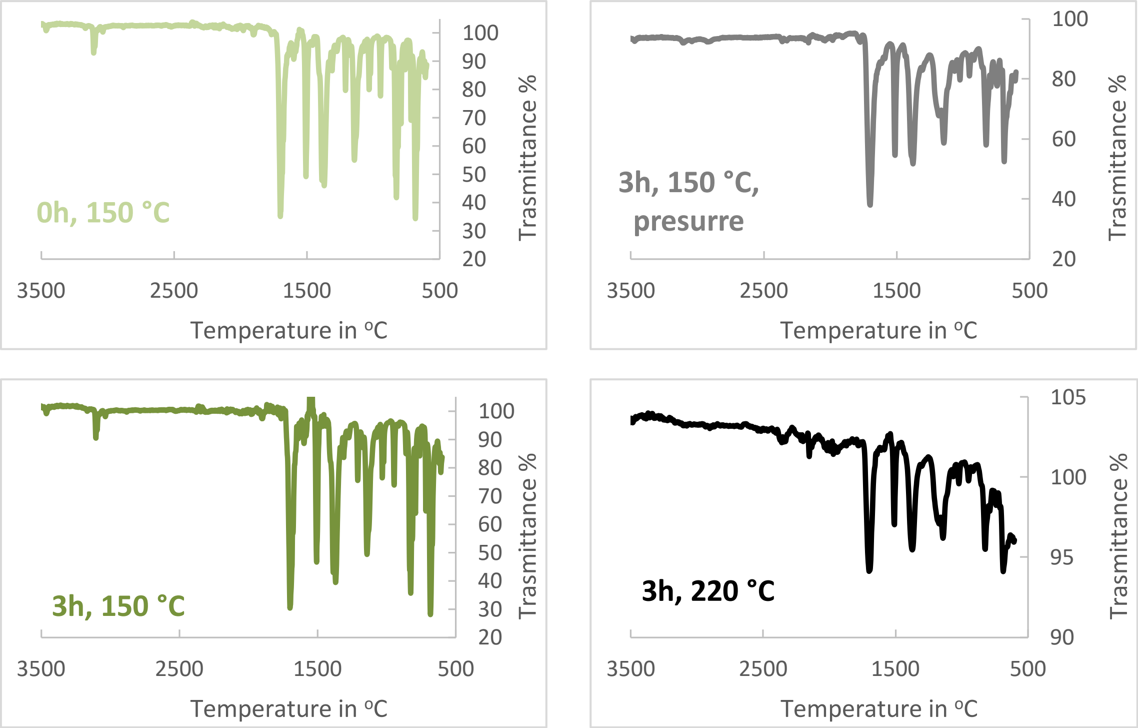

Figure S3. FTIR full spectra of MDP-BMI before and after 3 hours at 150 °C, after being in a press for 3 hours at 150 °C (4MPa), and after 3 hours at 220 °C.

Supplementary figures

Maleimide self-reaction in furan/maleimide-based reversibly crosslinked polyketones: Processing limitation or potential advantage?

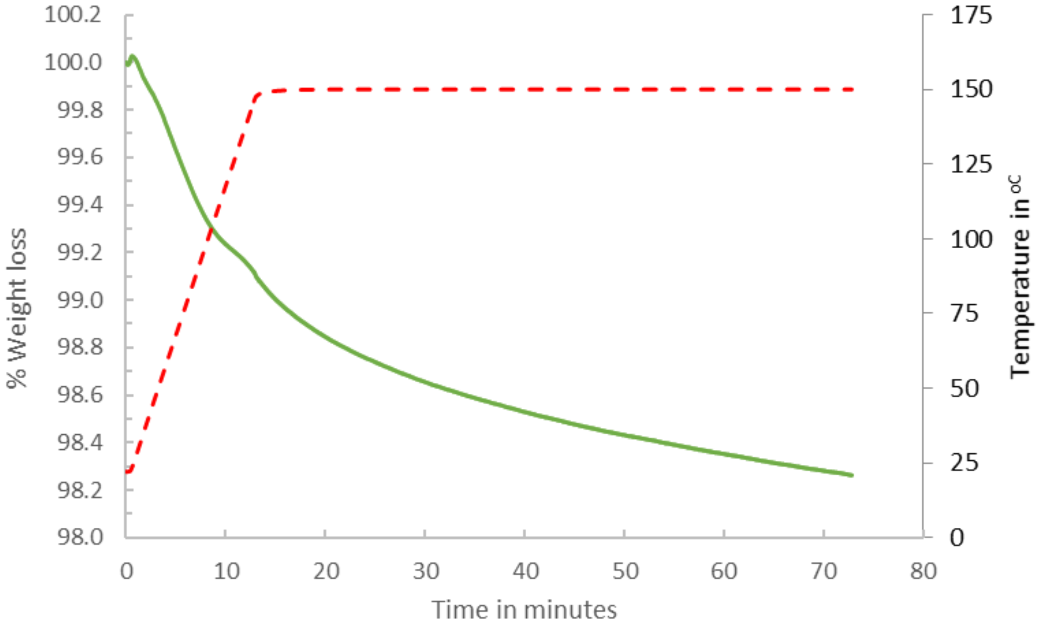

Figure S4. Thermo-gravimetric analysis of the polymer (weight loss percentage in green solid line and temperature in red dashed lines).

Supplementary figures

Maleimide self-reaction in furan/maleimide-based reversibly crosslinked polyketones: Processing limitation or potential advantage?

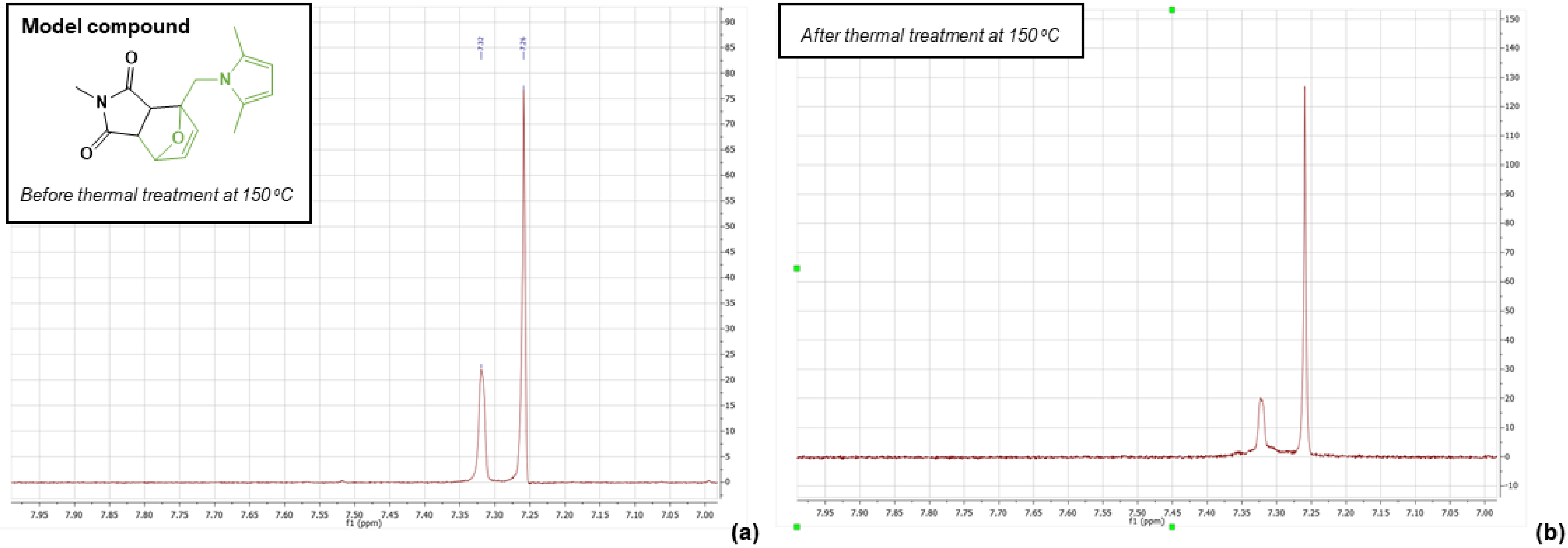

Figure S5. H[1]-NMR from 8 to 7 ppm of the model compound (a) before and (b) after the thermal treatment at 150 °C.
